# Perioperative outcomes and adverse events of robotic colorectal resections for inflammatory bowel disease: a systematic literature review

**DOI:** 10.1007/s10151-018-1766-5

**Published:** 2018-03-15

**Authors:** S. Renshaw, I. L. Silva, A. Hotouras, S. D. Wexner, J. Murphy, C. Bhan

**Affiliations:** 1 Department of Surgery, Whittington Hospital NHS Trust, London, UK; 2 National Centre for Bowel Research and Surgical Innovation, Queen Mary University of London, London, UK; 3 Department of Surgery, The Royal London Hospital, London, UK; 4 Digestive Disease Center, Cleveland Clinic Florida, Fort Lauderdale, FL USA; 5 Department of Surgery, Imperial College, London, UK

**Keywords:** Inflammatory bowel disease, Colorectal resection, Robotic surgical procedures

## Abstract

The purpose of this study was to assess outcome measures and cost-effectiveness of robotic colorectal resections in adult patients with inflammatory bowel disease. The Cochrane Library, PubMed/Medline and Embase databases were reviewed, using the text “robotic(s)” AND (“inflammatory bowel disease” OR “Crohn’s” OR “Ulcerative Colitis”). Two investigators screened abstracts for eligibility. All English language full-text articles were reviewed for specified outcomes. Data were presented in a summarised and aggregate form, since the lack of higher-level evidence studies precluded meta-analysis. Primary outcomes included mortality and postoperative complications. Secondary outcomes included readmission rate, length of stay, conversion rate, procedure time, estimated blood loss and functional outcome. The tertiary outcome was cost-effectiveness. Eight studies (3 case-matched observational studies, 4 case series and 1 case report) met the inclusion criteria. There was no reported mortality. Overall, complications occurred in 81 patients (54%) including 30 (20%) Clavien-Dindo III–IV complications. Mean length of stay was 8.6 days. Eleven cases (7.3%) were converted to open. The mean robotic operating time was 99 min out of a mean total operating time of 298.6 min. Thirty-two patients (24.7%) were readmitted. Functional outcomes were comparable among robotic, laparoscopic and open approaches. Case-matched observational studies comparing robotic to laparoscopic surgery revealed a significantly longer procedure time; however, conversion, complication, length of stay and readmission rates were similar. The case-matched observational study comparing robotic to open surgery also revealed a longer procedure time and a higher readmission rate; postoperative complication rates and length of stay were similar. No studies compared cost-effectiveness between robotic and traditional approaches. Although robotic resections for inflammatory bowel disease are technically feasible, outcomes must be interpreted with caution due to low-quality studies.

## Introduction

Laparoscopic colorectal resections are now routinely performed worldwide for patients with inflammatory bowel disease (IBD). Laparoscopy may be associated with less pain, reduced intra-abdominal adhesions, shorter duration of hospitalisation and quicker return to function compared to open surgery [1–3]. However, potential disadvantages include amplification of hand tremors and loss of wrist movement due to limited movement of the long instruments within the trocars [4]. Consequently, robotic platforms, such as the da Vinci^®^ system, have been developed in order to address these limitations. The theoretical advantages of these systems include a stable camera platform, three-dimensional image, excellent ergonomic function with ambidextrous capability and freedom of movement in multiple directions [5].

The first robotic colorectal procedure was performed in New Jersey, USA, in 2001 [6], and this approach has gained considerable support over the past 15 years, especially for pelvic surgery. Early studies demonstrated that robotic total mesorectal excision (TME) for rectal cancer was safe and feasible and achieved a low number of positive resection margins and low conversion rates [7, 8]. Indeed, early results from the first international, multi-centre randomised controlled trial of 471 patients comparing robotic with laparoscopic TME revealed similar oncological clearance, patient outcomes and conversion to open surgery rates [9]. Additionally, it has been proposed that it may reduce the risk of complications related to pelvic nerve injuries [10]. However, the advantages with respect to abdominal resections is less clear, as a randomised controlled study did not report any benefits associated with the use of robotic assistance compared with laparoscopic right hemicolectomy [11]. Furthermore, the expense of installing and maintaining these platforms is significant and potentially prohibits their widespread use, particularly in an environment of limited health care resources. For instance, a recent American study comparing robotic to laparoscopic colectomies found a mean cost increase of approximately $15,595 per case [12].

Evidence to justify the use of robotic colorectal resection in patients with IBD remains even sparser. Therefore, the aim of this study was to perform a systematic review of the published literature in order to report the clinical outcomes and cost associated with robotic colorectal resections for patients with IBD.

## Materials and methods

### Search strategy

**Fig. 1:**
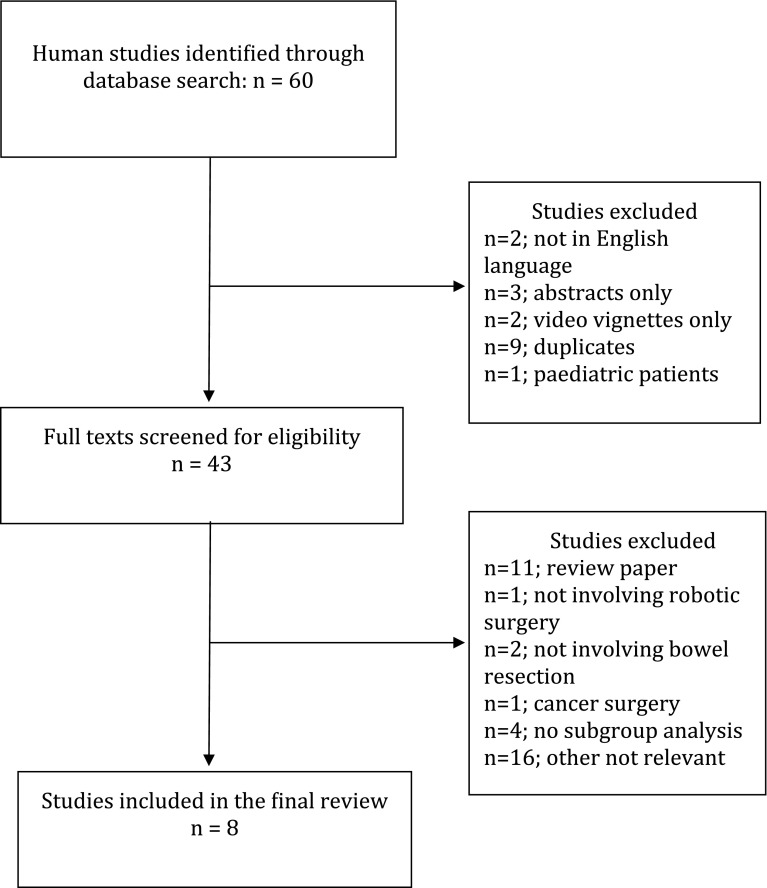
Diagrammatic illustration of search strategy

PubMed (1966–September 2016), Medline (1946–September 2016), the Cochrane Library and Embase (1947–September 2016) were electronically searched using the following text: “robotic(s)” AND (“inflammatory bowel disease” OR “Crohn’s” OR “ulcerative colitis”). In addition, reference lists of relevant articles, reviews and commentaries were manually searched, and experts in the specialty were contacted to identify papers not captured by electronic searches (Fig. 1). Studies searched were limited to those performed in adult humans and published in the English language. Furthermore, if the abstract or full manuscript was irrelevant or contained insufficient data (such as absence of subgroup analysis), it was excluded from the analysis.

### Study quality assessment

Quality assessment was performed using the National Institute for Health and Care Excellence (NICE), Quality Assessment for Case Series (QACS) tool [13] by scoring the studies out of a maximum of 8 points. A study scored 1 point each if; (a) it was multi-centre, (b) hypothesis/aim/objective clearly reported, (c) outcomes defined, (d) inclusion and exclusion criteria stated, (e) data prospectively collected, (f) patients consecutively recruited and (g) the main findings of the study are clearly described and (h) outcomes stratified.

### Study outcomes

The primary outcome measures of interest included mortality and postoperative complications, classified according to Clavien-Dindo grade I–IV [14]. Secondary outcome measures included: (i) readmission rates (ii) length of postoperative stay (LOS), (iii) conversion to open surgery rate, (iv) mean operating time, (v) estimated blood loss, (vi) functional outcomes. The tertiary outcome measure was cost-effectiveness, by comparing the cost of robotic procedures to its laparoscopic equivalent.

### Data extraction

Quantitative data were extracted by 2 independent reviewers (SR/AH), and results were cross-checked. Any discrepancies in results were resolved by repeat data extraction, discussion and further review of the index study.

### Data analysis

Given that the majority of studies lacked a control group, meta-analysis of the data was precluded. As such, the results from each study are presented in a summarised and aggregate form.

## Results

### Search results

The Preferred Reporting Items for Systematic reviews and Meta-Analyses (PRISMA) guidelines [15] were adhered to for the reporting of this systematic review. The electronic search yielded a total of 60 citations. Following examination of the full-text manuscript (*n* = 43), a final total of 8 studies were eligible for inclusion (Fig. 1). They included 150 patients; 10 with a diagnosis of Crohn’s disease, 2 with indeterminate colitis and 138 with ulcerative colitis (UC).

### Study characteristics and quality assessment

One of the 8 studies was a case report, 4 were case studies, and 3 were retrospective case-matched reviews comparing robotic to laparoscopic surgery in 2 studies and to open surgery in 1 study [16–23]. Quality assessment is recorded in Table 1.

**Table 1:** Summary of studies looking at robotic resections for inflammatory bowel disease

Authors Country, Year	Data collected	*n*	Pathology	Design	QA (out of 8)	Type of dissection	Technique	Single multiple surgeons	Procedure time (min) Mean ± SD	
									Robotic time	Total operative time
Byrn et al. [16] Iowa, USA 2014	Sept. 2010–Dec. 2012	18 (Period 1 = 12 vs. Period 2 = 6)	4:Crohn’s 14:UC	CS	4	Robotic proctectomy ± lap. colectomy ± lap. double-stapled IPAA + end ileostomy	4 robotic arms (camera port at the superior portion of umbilicus), assistant port in RUQ	Single	NR	Period 1 293.2 ± 95.7 vs. Period 2 264.7 ± 93.6
Mark-Christensen et al. [17] Aarhus, Denmark 2016	Jan. 2004–Sept. 2014	81 Control = 170	UC	CMOR	5	Robotic completion proctectomy ± lap colectomy (*n* = 2; the remaining 79 had previously undergone lap. Colectomy) + extracorporeal stapling of a J-pouch through a Pfannenstiel incision with formation of an IPAA using a circular stapler Control: open proctectomy + IPAA ± colectomy	Six trocars	Multiple	NR	284 ± 38 compared to open surgery 130 ± 38 *p* ≤ 0.01
McLemore et al. [18] CA, USA 2012	April 2010–June 2010	3	Toxic UC	CS	2	Robotic proctectomy with extracorporeal formation of J-pouch through a suprapubic incision and lap. assisted IPAA (lap. mobilisation of retroperitoneal small bowel mesentery) using circlar stapler, and diverting loop ileostomy Note variation in distal rectum transection—first two open, third using lap. stapler under robotic visualisation Note all patients had previously undergone lap. colectomy	4 robotic arms (RLQ, LLQ, LUQ, camera port in supraumbilical position), assistant SILS port at ileostomy site	Single	123 ± 14.9	436 ± 106.6
Miller et al. [19] IL, USA 2012	Jan. 2009–Aug. 2010	17 Control = 17	13:UC, 2: IC 2:Crohn’s	CMOR	4	10 lap. assisted (for mobilisation of distal ileal mesentery and division of superior haemorrhoidal artery) robotic proctectomy with extracorporeal formation of J-pouch through Pfannenstiel incision with formation of an IPAA through an open approach using a double-stapled technique, and loop ileostomy 7 laparoscopic-assisted (for division of superior haemorrhoidal artery) robotic completion proctectomy (CP) Control: Laparoscopic proctectomy Note all patients had previously undergone laparoscopic colectomy	3 robotic arms (RLQ and LLQ, 12 mm camera at umbilicus), assistant port at ostomy site, assistant port LUQ	NR	RP-CP: 90 ± 29.9 RP–IPAA: 86 ± 16.5	RP-CP: 351 ± 76.3 (compared to LP-CP *p* = 0.03) RP–IPAA: 370 ± 65.9 (compared to LP–IPAA *p* = 0.14)
Morelli et al. [20] Pisa, Italy 2015	Feb. 2010–March 2014	1	UC	CR	3	Robotic proctectomy following hand-assisted laparoscopic colectomy and subsequent extracorporeal formation of J-pouch, with hand-sewn IPAA via a perineal approach, and loop ileostomy	4 robotic arms	Single	99	238
Pedraza et al. [21] Tx, USA 2011	Aug. 2008–Feb. 2010	5	UC	CS	4	Robotic proctectomy following laparoscopic colectomy and subsequent extracorporeal formation of J-pouch and formation of IPAA under robotic vision, and loop ileostomy	4 robotic arms (12 mm camera at umbilicus, 8 mm RLQ, 8 mm LLQ, 8 mm L loin), 12 mm assistant port site R loin	Single	138.8 ± 35.0	330 ± 47.4
Rencuzogullari et al. [22] Cleveland, Ohio, USA 2016	Jan. 2010–June 2014	21 Control = 21	17 UC 4 Crohn’s	CMOR	2	Robotic completion proctectomy ± laparoscopic colectomy (*n* = 4) ± extracorporeal formation of J-pouch with formation of IPAA using circular stapler (*n* = 18) Control: laparoscopic completion proctectomy ± colectomy ± IPAA	Five to six ports	Multiple (5 surgeons)	NR	304 ± 109 (compared to 213 ± 86 for lap; *p* = 0.008)
Roviello et al. [23] Siena, Italy 2015	Jun. 2014–Dec. 2014	4	UC	CS	2	Robotic proctocolectomy with terminal ileostomy, lap-assisted for traction	3 robotic arms (RUQ, LUQ, 12 mm epigastrium camera), 1 12 mm LIF assistant port	NR	NR	235 ± *

Authors Country, Year	Conversion to open rate	Estimated Blood loss (ml)	Days to normal diet (days)	Return of bowel function (days)	Length of stay (days) Mean ± SD	Mean follow up (months)	Readmission	Complications following reversal of loop ileostomy	Costs (US dollars)	
Byrn et al. [16] Iowa, USA 2014	0	(Period 1) 252 ± 193 vs. (Period 2) 447 ± 367	(Period 1) 4.7 ± 2.9 vs. (Period 2) 3.0 ± 0.6	NR	(Period 1) 9.3 ± 5.3 vs. (Period 2) 5.3 ± 1.2	(Period 1) 11 (Period 2) 4.4	NR	NR	Observed: 19,278 ± 13,404 vs. 13,413 ± 2504 (not including cost of robot/maintenance)	
Mark-Christensen et al. [17] Aarhus, Denmark 2016	9 (= 11.1%)	NR	NR	NR	9.1 ± 5 compared to open surgery 11 ± 6.4 (*p* = 0.02)	3.35	32/81 (40%) compared to open surgery 44/170 (26%) (*p* = 0.03)	1/81 Pouch failure compared with open surgery: 2/170 pouch failures (*p* = 0.97)	NR	
McLemore et al. [18] CA, USA 2012	0	NR	NR	NR	11.7 ± 4.9	7.7	1/3 = 33% (for pulmonary embolism)	1/3 pouchitis	NR	
Miller et al. [19] IL, USA 2012	0	CP: 486 ± 295.4 (214 ± 244.5; *p* = 0.18) IPAA: 245 ± 136.3 (172 ± 143.1; *p* = 0.15)	NR	CP: 3.0 ± 0.8 (*p* = 0.04) IPAA: 3.6 ± 2.8 (*p* = 0.30)	CP: 6.4 ± 1.0 (*p* = 0.02) IPAA: 8.5 ± 3.8 (*p* = 0.17)	4.5	NR	Bowel movements: 6 per day ± 0.8 (*p* = 0.15) 5/6 night-time bowel movements (*p* = 1.0) 3/6 Spotting of stool or minor leakage (*p* = 0.58) 2/6 daytime pad use (*p* = 0.17) 2/6 night-time pad use (*p* = 0.54) 1/6 Anal pruritus (*p* = 0.14) 1/6 Able to postpone bowel movements (0.30) 3/6 weaker erection (*p* = 1.0)	NR	
Morelli et al. [20] Pisa, Italy 2015	0	57.5	NR	NR	15	20.3	NR	Bowel movements: 8	NR	
Pedraza et al. [21] Tx, USA 2011	0	200 ± 122.5	2 ± 0.6	2.4 ± 0.9	5.6 ± 2.6	NR	1/5 = 20%	NR	NR	
Rencuzogullari et al. [22] Cleveland, Ohio, USA 2016	2/21 = 9.5% (1 for unclear anatomy, 1 for extensive adhesions) (compared to 3/21 = 14.3% for lap., 1 for unclear anatomy, 1 for adhesions, 1 for bleeding; *p* ≥ 99)	360 ± 257 (compared to 188 ± 196 for lap; *p* = 0.002)	NR	2.29 ± 1.53 (compared to 2.79 ± 2.26 for lap; *p* = 0.62)	7.9 ± 6.4 (compared to 9.2 ± 7.5 for lap; *p* = 0.39)	* median recorded as 24 months (3–58)	3/21 = 14% (compared to 3/21 for lap; *p* = 1.0)	1/18 Pouch fistula (compared with lap. *p* ≥ 0.99) 1/18 anastomotic separation (compared with lap *p* ≥ 0.99)	NR	
Roviello et al. [23] Siena, Italy 2015	0	100 ± *	NR	* Median 3 days	* Median 6 days	NR	NR	NR	NR	

*CD* Clavien-Dindo, *CMOR* case-matched observational, retrospective data collection, *CP* completion proctectomy, *CR* case report, *CS* case series, *IC* indeterminate colitis, *IPAA* Ileal pouch-anal anastomosis, *lap* laparoscopic, *LLQ* left lower quadrant, *LUQ* left upper quadrant, *NR* not reported, *QA* quality assessment, *RLQ* right lower quadrant, *RUQ* right upper quadrant, *TPN* total parenteral nutrition, *UC* ulcerative colitis

*Results not fully presented in paper. Results are presented as a mean ± SD unless specified to be a median, where the results are represented as median (range)

### Type of procedure performed

The type of procedure performed in each of the studies varied as well as the extent of robotic involvement (Table 1). The most extensive involvement of the robotic system was used by Roviello et al. [23] in 4 patients involving a single-docking robotic proctocolectomy with formation of a terminal ileostomy. A conventional laparoscopic port was also used during these procedures, principally to assist with additional traction. The remaining studies performed robotic proctectomy with or without variable amounts of laparoscopic assistance ± laparoscopic colectomy ± extracorporeal formation of a J-pouch with formation of a hand-sewn/stapled (open or laparoscopic) ileal pouch-anal anastomosis (IPAA) via a perineal/Pfannenstiel/suprapubic incision ± diverting loop ileostomy (Table 1). Further classification regarding the exact type of procedure for each patient was not recorded in every study [16].

### Primary outcomes

#### Mortality

There was no mortality in any of the studies.

#### Postoperative complications

Complications occurred in 81 patients (54%) including serious complications (Clavien-Dindo grade III–IV) in 30 patients (20%). Early postoperative morbidity varied from 0 to 100% (Table 2). 100% of the high-risk patients operated on with fulminant UC by McLemore et al. [18] experienced early complications, although all complications were a maximum of Clavien-Dindo II. The single case report did not describe any complications [20]. The remaining studies reported a morbidity rate of 40–75%. Rencuzogullari et al. [22] reported no statistically significant difference between postoperative complications following robotic and laparoscopic approaches, with similar postoperative outcomes reported in the second case-matched study (although no *p* value was obtained) [19]. The single case-matched retrospective study comparing robotic to open proctectomy also reported no statistically significant differences in postoperative complications between the two groups [17]. In this study, 10% of the patients undergoing robotic surgery underwent a reoperation, which was statistically similar to the 5% in the control group (*p* = 0.18).

**Table 2:** Table of postoperative complications

Authors	Method of adverse event ascertainment	Early postoperative complications in robotic group			Early postoperative complications in control group		
		Clavien-Dindo I–II	Clavien-Dindo III–IV	Overall	Clavien-Dindo I–II	Clavien-Dindo III–IV	Overall
Byrn et al. [16]	NR	6/18 = 33.3% 5/18 superficial surgical site infection (Period 1; 4 vs. Period 2; 1), 1/18 required blood transfusion	Grade IIIa/b = 4/18 = 22.2% 4/18 deep surgical site infection and organ space infections e.g. anastomotic leak or pelvic abscess requiring IR drainage (Period 1; 3 vs. Period 2; 1)	10/18 = 55.6%	N/A	N/A	N/A
Mark-Christensen et al. [17]	NR	15 grade I complications 23 grade II complications (complications not specified)	4 grade IIIa complications 14 grade IIIb complications 1 grade IVa complications 6/81 Bleeding 2/21 Anastomotic leak 1/81 Pouch leak 4/81 Intra-abdominal abscess 1/81 Abdominal wall abscess 1/81 Mechanical bowel obstruction 2/81 Anastomotic stenosis 1/81 Pouch fistula 1/81 Myonecrosis of lower extremity	57 complications overall, occurring in 40 patients = 40/81 = 49.4% 41 patients experienced no complications	OPEN SURGERY 20 grade I complications (*p* > 0.15) 44 grade II complications (*p* = 0.67) (complications not specified)	OPEN SURGERY 4 grade IIIa complications (*p* > 0.28) 17 grade IIIb complications (*p* = 0.10) 0 grade IVa complications (*p* = 0.15) 2/170 bleeding 2/170 perianal abscess 3/170 intra-abdominal abscess 2/170 intra-abdominal haematoma 2/170 fascia dehiscence 1/170 anastomotic necrosis 1/170 parastomal hernia 1/170 pneumothorax 2/170 pouch fistulas 5/170 anastomotic stenosis	OPEN SURGERY 85 complications overall in 65 patients = 65/170 = 38.2% 105 patients experienced no complications (*p* = 0.09)
McLemore et al. [18]	NR	3/3 = 100% 1/3 high output ileostomy 1/3 pelvic abscess (treated with antibiotics), urinary retention, ileus (TPN) PE 1/3 ileus (TPN)	0/0 = 0%	3/3 = 100%	N/A	N/A	
Miller et al. [19]	NR	9/17 = 52.9% 2/17 partial small bowel obstruction—non-operative management 1/17 ileus 1/17 superficial surgical site infection 1/17 pulmonary infection 4/17 urinary infection	CDIIIa/b = 2/17 = 11.8% 1/17 anastomotic leak—IR drainage 1/17 incisional hernia	11/17 = 64.7%	LAP. SURGERY 9/17 = 52.9% 1/17: ileus 2/17 wound infection 3/17 urinary retention 3/17 urinary infection	LAP. SURGERY CD III/IV = 4/17 = 23.5% 2/17 anastomotic leak 2/17 incisional hernia)	LAP. SURGERY 13/17 = 76.5%
Morelli et al. [20]	NR	0/1 = 0%	0/1 = 0%	0%	N/A	N/A	
Pedraza et al. [21]	NR	1/5 = 20% 1/5 Ileus and dehydration	CD III = 1/5 = 20% 1/5 presacral collection (IR drainage)	2/5 = 40%	N/A	N/A	
Rencuzogullari et al. [22]	NR	9/21 = 42.9% 5/21 = ileus 1/21 = Urinary retention 1/21 = DVT 2/21 = Pneumonia	CD III = 3/21 = 14.3% 3/21 = Organ space surgical site infection necessitating drainage under anaesthesia	12/21 = 57.1%	LAP. SURGERY 9/21 = 42.9% 4/21 = ileus (*p* = 0.73) 2/21 = wound infection (*p* = 0.11) 2/21 = Urinary retention (*p* > 0.99) 1/21 = DVT (*p* ≥ 0.99) 0/21 = Pneumonia (*p* = 0.49)	LAP. SURGERY CD III/IV = 1/21 = 4.8% 1/21 = Organ space surgical site infection, drainage under anaesthesia (*p* = 0.61)	LAP. SURGERY 10/21 = 47.6%
Roviello et al. [23]	NR	2/4 = 50% 1/4 superficial surgical site infection 1/4 acute pulmonary oedema	CDIIIb = 1/4 = 25% 1/4 Small bowel obstruction secondary to internal hernia—re-laparoscopy with no bowel resection	3/4 = 75%	N/A	N/A	

*IR* interventional radiology, *lap* laparoscopic, *NR* not recorded

### Secondary outcomes

#### Readmission rates

Overall, 37 patients (24.7%) were readmitted. Mark-Christensen et al. [17] reported a readmission rate of 40%, which is a significantly higher rate than that seen in the open surgery group (*p* = 0.02 on univariate analysis and *p* = 0.03 on multivariate analysis). Rencuzogullari et al. [22] reported no significant difference in readmission rate between the robotic and laparoscopic group (*p* = 1.0).

#### Length of stay (LOS)

Overall, the mean LOS for 146 patients was 8.6 days, ranging from 5.3 ± 1.2 to 15 days (Table 1). The 4 patients in the study by Roviello et al. [23] were excluded as only a median LOS was reported. Mark-Christensen et al. [17] reported a mean LOS of 9.1 ± 5 days following robotic surgery which was significantly shorter than the 11 ± 6.4 days following open surgery (*p* = 0.02), however in multivariate regression analysis taking into account the primary operation, body mass index, American Society of Anaesthesiologists classification, sex and age this finding was no longer significant (*p* = 0.07). Rencuzogullari et al. [22] reported a mean LOS of 7.9 ± 6.4 days which was not statistically significant from their laparoscopic group (*p* = 0.39). Miller et al. [19] also reported no significant difference in mean LOS between colectomised patients who underwent robotic proctectomy (RP) + IPAA (RP–IPAA) (8.5 ± 3.8 days) and those who underwent laparoscopic proctectomy (LP) + IPAA (LP–IPAA) (6.1 ± 2.2 days; *p* = 0.17); however, there was a significantly longer mean LOS for robotic completion proctectomy (RP-CP) (6.4 ± 1.0 days) compared with laparoscopic completion proctectomy (LP-CP) (4.1 ± 0.7 days; *p* = 0.02).

#### Conversion rates and estimated blood loss (EBL)

Overall, 11 robotic procedures were converted to open (7.3%). Six studies reported a conversion rate of zero, including analysis of 48 patients. The remaining 2 studies [17, 22] reported a conversion rate of 11.1 and 9.5%, respectively. Reasons for conversion in reported by Rencuzogullari et al. [22] included 1 case of extensive adhesions and 1 case of unclear anatomy. There was no significant difference in conversion rate between robotic and laparoscopic approaches [22].

EBL was recorded in 6 studies (Table 1), involving 66 patients, with a wide variation: 57.5–486 ml [19, 20]. Overall, mean (median of the means) EBL was 248.5 ml. Of note, Miller et al. [19] did not show a significant difference in EBL between RP-CP and LP-CP (*p* = 0.18), or between RP–IPAA and LP–IPAA (*p* = 0.15). However, Rencuzogullari et al. [22] reported a significantly higher EBL for the robotic compared to laparoscopic approach (*p* = 0.002).

#### Procedure time

Direct comparison of the reported procedure time was difficult due to the heterogeneity of procedures between the studies (Table 1). Additionally, some studies only reported total operative time rather than a robotic time. However, the overall mean robotic operating time (median of the means) was 99 min out of a mean total operative time of 298.6 min.

McLemore et al. [18] reported a mean ± SD robotic time of 123 ± 14.9 min out of a total procedure time of 436 ± 106.6 min. This total procedure time is notable, especially considering that all patients had previously undergone laparoscopic colectomy. However, this cohort underwent surgery for acute, severe disease with a diagnosis of fulminant UC. Roviello et al. [23] reported the shortest total procedure time 235 min and interestingly performed the most robotic surgery involving robotic colectomy in addition to proctectomy, although no anastomosis was performed.

Miller et al. [19] directly compared total procedure time ± SD for colectomised patients undergoing RP-CP (351 ± 76.3 min) to LP-CP (238 ± 66.4 min), and there was a significant time increase with robotic procedures (*p* = 0.03). However, this difference was not significant for colectomised patients undergoing RP–IPAA (370 ± 65.9 min) compared to LP–IPAA (316 ± 78.4 min; *p* = 0.14). Rencuzogullari et al. [22] reported a mean total operative time ± SD of 304 ± 109 min for robotic proctectomy ± laparoscopic colectomy ± IPAA which was significantly longer than the equivalent laparoscopic procedure (213 ± 86 min; *p* = 0.008). Finally, as would be expected, Mark-Christensen et al. [17] reported a significantly longer total operative time ± SD for robotic proctectomy ± laparoscopic colectomy + IPAA (284 ± 38 min) compared to the equivalent open procedure (130 ± 38 min), which was significant in both univariate and multivariate analysis (*p* = <0.01).

#### Functional outcomes

The 3 case-matched studies reported similar long-term pouch outcomes between robotic and laparoscopic or open procedures. Mark-Christensen et al. [17] reported 1 episode of pouch failure in the robotic group (*p* = 0.97), whilst Rencuzogullari et al. [22] reported 1 episode of pouch fistula (*p* > 0.99) and 1 episode of anastomotic separation (*p* > 0.99). Miller et al. [19] reported similar functional outcomes between RP–IPAA and LP–IPAA after reversal of ileostomy. Although the numbers are small, there was no significant difference in pouch function and continence, specifically anal continence during daytime and night time (*p* = 0.58), minor leakage (*p* = 0.58), frequency of bowel movements (*p* = 0.15), ability to postpone bowel movements (*p* = 0.30) and anal pruritus (*p* = 0.14) between these cohorts. Post-procedure quality of life scores (*p* = 1.0), as well as sexual functional outcome measures including change in sexual desire (*p* = 0.66) and quality of erection (*p* = 1.0), were equivalent in the 2 groups [19].

Mean return of bowel function in days, following robotic proctectomy, was reported in 3 studies. Rencuzogullari et al. [22] reported a mean ± SD of 2.3 ± 1.5 days equivalent to that seen in its laparoscopic group (*p* = 0.62). This length was comparable to mean return of bowel function reported by Miller et al. [19] for RP-CP (3.0 ± 0.8 days) and RP–IPAA (3.6 ± 2.8 days). Although return of bowel function following RP-CP took significantly longer than after LP-CP (*p* = 0.04), it was statistically equivalent for RP–IPAA and LP–IPAA (*p* = 0.3). Pedraza et al. [21] reported a mean ± SD of 2.4 ± 0.9 days to return of bowel function following robotic proctectomy + laparoscopic colectomy + IPAA, which was broadly equivalent to the other 2 studies. Mean number of days to normal diet was reported in 2 studies. Pedraza et al. [21] reported a mean of 2 ± 0.6 days which was comparable to that reported by Byrn et al. (4.7 ± 2.9 and 3.0 ± 0.6) [16].

### Tertiary outcomes

#### Cost implications

Only 1 study reported a cost analysis [16]. Direct costs observed for robotic IBD cases, excluding cost of acquisition, depreciation, amortisation and maintenance of the robotic platform, showed a decreasing trend over a period of 27 months ($19,278 ± 13,404 vs. $13,413 ± 2504; *p* = 0.06). The ratio of observed to expected cost, which aims to correct for patient-specific factors that increase cost outside of the surgical procedure, decreased over time (1.8 ± 0.8 vs. 1.2 ± 0.1; *p* = 0.02) perhaps as a result of decreased operating time and length of stay. No study compared the cost of robotic surgery to traditional approaches.

## Discussion

This systematic review identified 8 studies reporting outcomes following robotic colorectal resection in 150 patients with IBD. Notably, there were no randomised trials; however, 2 retrospective studies showed comparable results between robotic and laparoscopic surgery and 1 between robotic and open surgery. The studies were heterogeneous in terms of the populations studied, procedures performed and criteria by which outcomes were measured, precluding formal meta-analysis.

### Primary outcomes

The rate of reported pelvic sepsis, arguably the most significant early complication following IPAA, in each of the studies analysed in this review fell within the range reported following IPAA in a meta-analysis of 43 observational studies (range 2.3–26.7%) [24]. However, the overall morbidity rate of 54% is higher than recent published literature evaluating laparoscopic surgery for IBD (Table 3) [25–34]. Although most studies defined the morbidity rate as the number of patients affected by complications [18, 20, 21, 23, 25–29, 31, 32, 34], several studies, particularly those in this review [16, 19, 22], did not specify how many patients were affected.

**Table 3:** Recent studies reporting laparoscopic rectal dissection in patients with inflammatory bowel disease and their associated perioperative outcomes

Authors	Study type	Data collected	Disease, operation	No. cases	Mortality %	Morbidity	Readmission rate	LOS (Days) Mean ± SD	Conversion rate	Procedure time (min) Mean ± SD	EBL (ml) Mean ± SD	Functional outcome
El-Gazzaz et al. [25] Ohio, USA [24]	CMOR	1992–2007	IBD/FAP, proctectomy ± colectomy + IPAA	119	0%	23.1%	NR	Median 5 (4–7)	7.6%	Median 272 (231–332)	Median 250 (150–400)	Median time first BM: 2 (2–3); BMs/day (mean ± SD) 4.9 ± 2.2: BMs/night 1.7 ± 1.4; continence 94%
Lefevre et al. [26] Paris, France [25]	Observational, prospective	2002–2008	IBD/FAP, elective proctectomy ± colectomy + IPAA	82	0%	32%	22%	13	11%	288	NR	BMs/day: (mean ± SD) 6 ± 3: BMs at night: 1 ± 1
Fichera et al. [27], Chicago IL, USA [26]	CMOP	2002–2007	UC, elective proctectomy ± colectomy + IPAA	73	1.4%	NR in full, however septic complications reported in 19.2%	NR	8.3	1.4%	335.4	231.5	BMs per day: 6.8 ± 2.8 (Mean ± SD); Mean days to normal diet: 5.5 Mean days to first BM: 4.8
Fajardo et al. [28], St Louis, MO, USA [27]	CMOR	1999–2008	UC, Proctocolectomy (± hand assistance) + IPAA	55	NR	50.1%	NR	8.4 ± 6	NR	266.7 ± 55	294 ± 274	Mean length of return of BM: 4.9 days ± 4.9
Fleming et al. [29], NY, USA [28]	CMOR	2005–2008	UC/FAP, proctocolectomy + IPAA	339	0.3%	26.8%	NR	7.3 ± 4.3	NR	124	NR	NR
Dolejs et al. [30], Wisconsin, USA [29]	CMOR	1998–2008	UC, elective/urgent lap-assisted proctocolectomy + IPAA	100	NR	NR in full, but SBO reported in 21%	NR	6.4 ± 3.3	NR	434 ± 73	285 ± 202	NR
Goede et al. [31], Reading, UK [30]	Prospective, observational	1999–2010	UC, lap-assisted proctectomy ± colectomy	72	0%	32%	10%	Median 7 (2–64)	7%	Median 210 (75–330)	NR	Median time to diet 36 h (4–168); Median BMs/day: 4 (2–8); Continence 90%; able to defer BMs: 98%; Failure (excision/diversion): 2.7%; pouchitis 0%
Duff et al. [32], Leeds UK [31]	Prospective observational	2006–2010	UC/FAP, Elective proctectomy ± colectomy + IPAA	75	0%	24%	24%	Median 7 (2–62)	0%	NR	NR	Continence diurnal: 87%: nocturnal 77%; Median (range) BMs per day: 7 (4–7): per night 1(0–2); pouchitis 24%
Schiessling et al. [33], Heidelberg, Germany [32]	RCT	2004–2008	UC/FAP, proctocolectomy +IPAA	21	NR	28%	NR	12	23.8%	313	261.5 ± 195.4	NR
Inada et al. [34], Japan [33]	CMOR	2004–2014	UC, elective proctocolectomy ± IPAA	12	0%	41.7	0%	Median 22.5 (12–35)	0%	Median 415 (258–546)	Median 415 (258–546)	1/12: pouchitis
This review	Systematic review	2004–2014	IBD, robotic proctectomy ± lap colectomy ± IPAA	150	0%	54%	24.7%	8.6	7.3%	298.6	248.5	

Results are presented as a mean ± SD unless specified to be a median, where the results are represented as median (range)

*BMs* bowel movements, *CD* Clavien-Dindo, *CMOP* case-matched observational prospective, *CMOR* case-matched observational retrospective, *FAP* familial adenomatous polyposis, *IPAA* ileal pouch-anal anastomosis, *NR* not recorded, *SBO* small bowel obstruction, *UC* ulcerative colitis

There was no significant difference in early postoperative complications between robotic and open surgery following IPPA [17], largely consistent with early meta-analyses of laparoscopic versus open studies during the ascending phase of the laparoscopic learning curve which reported, aside from a lower incidence of wound infection in laparoscopic surgery [35], equivalent adverse event rates between the 2 groups [35–37]. More recent studies indicate that laparoscopic surgery is associated with fewer early complications and lower rates of pelvic sepsis [39, 40] than open surgery [28, 33, 38]. However, the supplementary use of mini-laparotomies or Pfannenstiel incisions in robotic surgery is often necessary and may limit the benefits that have been seen in other minimally invasive surgical procedures. In the study by Mark-Christensen et al. [17], rectal stapling was performed using non-endoscopic staplers in one-third of the robotic cases, to ensure an adequate level and completeness of the stapling, emphasising an important technical challenge to this approach [17].

The case-matched robotic and laparoscopic studies [19, 22] also showed comparable morbidity rates, although 1 study did not analyse *p* values [19]. Studies evaluating the adoption of robotic technology for rectal cancer patients suggest 3 phases, with the first phase of learning achieved within a range of 9–41 cases [41–43]. It is likely, therefore, that the surgeons in these case-matched studies were in their initial learning curve of robotic surgery, though masters in laparoscopy and yet, robotic surgery provided comparable outcomes [17, 22, 29]. The absence of randomised controlled trials, however, makes any definitive conclusions difficult. Furthermore, the ROLARR trial has not shown any reduction in 30-day morbidity in robotic compared to laparoscopic rectal cancer resections [9].

### Secondary outcomes

Overall secondary outcomes were consistent with published laparoscopic studies (Table 3) [25–34].

Robotic readmission rates were similar to rates reported in laparoscopic case-matched controls [19, 22] although higher than in open surgery [17]. Importantly, this was not reflected in a higher major complication rate on readmission, indicating that there may be a lower threshold to readmit trial patients undergoing minimally invasive surgery [17]. Of note, the period during which readmission was analysed was not always declared and as such may represent a source of bias.

LOS was statistically similar to case-matched laparoscopic and open studies [17, 22]. Miller et al. [19] proceeded with RP-CP, in order to establish and optimise robotic surgical technique and to avoid affecting long-term functional outcomes associated with restorative procedures, which may go some way to explain the better results they achieved with RP–IPAA as they had accumulated more experience. This is supported by Byrn et al. [16] who reported a significant reduction in mean LOS following robotic proctectomy over a 2-year period (*p* = 0.03*)*, suggesting that recovery after robotic surgery may be quicker when performed by experienced operators. Of note, temporal and spatial patterns in adherence to principles of fast-track surgery may represent a substantial source of bias; the paper with the largest number of patients collected data over a 10-year period, from 2004 to 2014 [17].

Although there was no significant difference in conversion rates between case-matched robotic and laparoscopic procedures [22], it is important to note that conversion rate is a subjective endpoint. Overall EBL was consistent with EBL in laparoscopic studies (Table 3).

Procedure time was significantly longer than the time reported for case-matched laparoscopic [22] and open procedures [17]; however, the overall operative time appears comparable to published laparoscopic literature (Table 3). Conceivably, the operative time may decrease with increasing experience as was seen in the study by Miller et al. [19] where later procedures (RP–IPAA) were not significantly longer than their laparoscopic equivalent. Additionally, Byrn et al. [16] and Mark-Christensen et al. [17] showed a trend towards a decrease in total procedure time for robotic procedures over the course of their studies, although it was not clear at what stage of the procedure the time was shortened and the improvement was not statistically significant. Newer platforms, or hybrid procedures involving laparoscopic with robotic techniques, may help to reduce the necessity of multiple docking and operative times [22].

The ultimate purpose of IPAA surgery is to ensure satisfactory pouch function, which is an outcome that many papers did not assess. Heterogeneity of functional outcome measured makes comparison difficult; however, the case-matched studies showed no difference in days to return of bowel function following robotic or laparoscopic IPAA [19, 22] or pouch failure rates [17, 22]. One study reported no differences in pouch function, quality of life and sexual function after robotic or laparoscopic procedures [19]. It is worthwhile noting that the majority of patients with IBD are young and therefore future studies should include functional, including sexual, data analysis.

### Tertiary outcomes

The overall economic feasibility of robotic surgery for IBD was not determined, as only 1 study, without a control group, assessed the cost of robotic surgery [16]. Currently, it seems likely that high costs will prevent widespread adoption of robotic surgery in the near future, particularly without any evidence of improved outcomes.

### Limitations

In addition to those limitations already discussed, the most important limitation of this review is the low-quality papers analysed, mainly observational, single-centre, single-surgeon, retrospective, non-randomised designs with low patient numbers. Additionally, the analysed papers did not have subgroup analyses, which made comparing results for one specific operation or a specific disease (e.g. Crohn’s or UC) impossible. As such, patients with different diseases, preoperative conditions and operations were compared, leading to bias when comparing postoperative outcome. Finally, analysing articles in the English language only has limited the coverage of the review.

## Conclusions

Outcome data of robotic surgery for IBD must be interpreted with caution due to low-quality studies. However, robotic resections in patients with IBD are technically feasible. The significantly higher overall costs necessitate evidence for advantages over traditional approaches. Thus far, no such advantages have been demonstrated precluding a recommendation for widespread adoption.
